# Physical activity and mood in daily life – a multi-burst ambulatory assessment study disentangling state and trait components of within-person associations

**DOI:** 10.1186/s12966-026-01932-x

**Published:** 2026-05-14

**Authors:** Robin Olfermann, Iris Reinhard, Anastasia Benedyk, Oksana Berhe, Johanna Rehder, Marco Giurgiu, Urs Braun, Ulrich Ebner-Priemer, Heike Tost, Andreas Meyer-Lindenberg, Markus Reichert

**Affiliations:** 1 Department of Sport and Exercise Science, Research Group Sport and Exercise Psychology, Paris Lodron University Salzburg, Salzburg, Austria; 2 Faculty of Sport Science, Junior Research Group eHealth and sports analytics, Ruhr University Bochum, Bochum, Germany; 3 Department of Sports and Sports Science, Mental mHealth Lab, Karlsruhe Institute of Technology, Karlsruhe, Germany; 4 Department of Biostatistics, Medical Faculty Mannheim, Central Institute of Mental Health, Heidelberg University, Mannheim, Germany; 5 Department of Psychiatry and Psychotherapy, Medical Faculty Mannheim, Central Institute of Mental Health, Heidelberg University, Mannheim, Germany; 6 Institute of Movement Therapy and movement-oriented Prevention and Rehabilitation, German Sport University Cologne, Cologne, Germany

**Keywords:** Ambulatory assessment, Ecological momentary assessment, Wearables, Accelerometry, Non-exercise activity, Affective well-being, Traits, States

## Abstract

**Background:**

Physical activity (PA) and mood are associated in daily life. Prior studies showed that these associations are moderated by contextual factors (e.g., weather conditions) and differ between individuals. Yet, it remains unclear to what extent associations between PA and mood reflect stable, person-specific characteristics or fluctuate over time.

**Methods:**

We examined the temporal stability of within-person PA–mood associations derived from three one-week ambulatory assessments across three consecutive years in 65 adolescents and young adults. Mood was assessed via e-diaries, and PA was recorded with accelerometers. Multilevel models estimated the effects of preceding PA on mood. Between-person differences and within-person stability were quantified by random-effect variances and correlations across participants and weeks.

**Results:**

As hypothesized, PA was positively associated with subsequent energetic arousal (β = 4.88, *p* < .001) and valence (β = 1.27, *p* = .042) but negatively with calmness (β = −1.86, *p* = .003). Intraclass Correlation Coefficients indicate that 64%, 44%, and 80% of variance of the weekly PA-mood associations of individuals are attributable to between-person differences, while 36%, 56%, and 20% of variance are accounted for by within-person fluctuations in associations of PA with energetic arousal, valence, and calmness, respectively.

**Conclusions:**

For the first time, we show that PA–mood associations reflect a combination of both stable ‘trait’ components (between subjects) and ‘state’ variability (within subjects). Both have important implications for personalized and context-sensitive intervention approaches, such as just-in-time adaptive interventions. Future research is needed to unravel which situational components and person characteristics shape the variability of associations.

**Supplementary Information:**

The online version contains supplementary material available at 10.1186/s12966-026-01932-x.

## Background

Physical activity (PA) is widely recognized as a key factor in promoting physical and mental health [1–3]. In particular, its association with affective phenomena (e.g., mood) has been well documented [4–6]. Whereas earlier research on the effects of PA on mood was predominantly conducted in laboratory settings or relied on between-subject designs, recent years have seen a growing use of ambulatory assessment (AA) methods to study these associations in daily life [6]. AA is often referred to as ecological momentary assessment (EMA) or as experience sampling method (ESM), although definitions may vary [7]. It combines tools such as activity trackers and smartphone-based questionnaires (e-diaries) to capture behavior and experiences in real time and naturalistic contexts [8]. These methods offer a number of advantages, including real-time collection of ecologically valid data in naturalistic contexts, the ability to capture dynamic fluctuations of variables that naturally vary over time, such as mood or PA, the possibility to separate within- from between-person effects, and the collection of diverse physiological, behavioral and contextual data using multimodal sensors [7, 9].

A growing body of AA studies has demonstrated significant within-subject associations between PA and mood in daily life [6], demonstrating that higher levels of PA are typically followed by more positive mood in daily life. Conversely, studies have shown that sedentary behavior is negatively linked to momentary mood [10, 11]. However, the strength of associations between PA and mood varies considerably across studies [6]. The variability of effect sizes may result from a wide range of factors. These include person-specific characteristics (e.g., biological and lifestyle factors), within-subject variability (e.g., hormonal fluctuations, contextual influences, such as weather or season), and methodological differences between studies (e.g., mood assessments, PA quantification, heterogeneity of analyses methods).

While PA is positively associated with mood in daily life in general, research also shows that the strength of these associations differs between individuals [12, 13]. In addition to this variation between individuals, differential effects have been reported depending on the type of activity performed and the specific mood outcome investigated [14–16]. For example, contextual factors, such as weather conditions, have been found to moderate these associations [17].

Taken together, evidence indicates that PA–mood associations differ both between and within individuals across situations and contexts. However, no study to date has examined the extent to which within-person associations of PA and mood relate to stable characteristics of individuals or fluctuate within persons over time. In personality psychology, it is well established that individual experiences and behaviors result from both stable, person-specific ‘traits’ and situational, momentary ‘states’ [18]. Similarly, PA–mood associations may also reflect a combination of trait-like stability and state-like variability: some individuals may consistently benefit more from PA than others, while situational factors determine whether and to what extent these benefits occur in daily life.

To investigate this question empirically, the present study examines the temporal stability of within-person PA–mood associations derived from one-week of ambulatory assessments per year across three consecutive years in a sample of adolescents and young adults. This age group is characterized by pronounced developmental changes, providing a particularly salient context to examine the stability versus variability of within-person PA–mood associations [19, 20]. For instance, pubertal maturation [21], ongoing development of affect regulation and reward processing [22], and shifts in sleep–wake timing [23] can affect mood variability and affective responses to physical activity. Moreover, prominent contextual changes during this period, such as transitioning from school to university or work, increasing autonomy, and changes in social and physical environments, can reshape when, where, and with whom PA occurs, influencing both PA patterns and within-person associations between PA and mood [15, 24, 25]. Specifically, we aimed to examine whether individuals’ PA–mood associations remain stable or change over the course of three years and to what extent the effects vary between versus within individuals over time.

Based on previous findings on the associations of PA and mood in everyday life [6], we hypothesize that daily life PA will be positively related to momentary energetic arousal (H1) and valence (H2) and negatively to calmness (H3). Building on the idea that there may be a trait-like component shaping individuals’ responses to PA, we further expect meaningful between-person differences in these effects (H4), which we anticipate to be correlated across the three years (H5).

## Methods

### Participants

A total of 619 adolescents and young adults were recruited by the Psychoepidemiological Center at the Central Institute of Mental Health (CIMH) to participate in a seven-day ambulatory assessment study between September, 2014, and October, 2020. Participants were randomly selected from the local population registry of the city of Mannheim, using a two-stage stratified sampling procedure that accounted for population characteristics such as age, sex, and nationality. The presence of a psychiatric diagnosis, as assessed by the Mini-DIPS3 or SCID-IV, served as an exclusion criterion (for details, see [26, 27]). All participants provided written informed consent in accordance with the protocol approved by the institutional review board of Heidelberg University and received monetary compensation for their participation.

A subsample of 92 participants took part in two additional one-week follow-up assessments approximately one and two years after the initial assessment (on average 11.6 months between assessments 1 and 2, and 12.7 months between assessment 2 and 3). From this group, we excluded all participants who met any of the following criteria in at least one of the three assessment weeks: missing e-diary or accelerometry data (*n* = 14), defective accelerometry data (*n* = 12), fewer than 15 completed e-diaries per week (*n* = 1), or e-diary compliance below 30% (*n* = 0). The ≥ 15 e-diaries criterion was chosen as a pragmatic rule of thumb [28] to ensure stable estimation of participant- and week-specific within-person slopes and minimize shrinkage-related bias. Compliance was calculated as the percentage of answered e-diaries relative to the total number of prompted e-diaries. This resulted in a final sample of 65 participants with complete seven-day ambulatory assessment data across three consecutive years, collected between November 2014 and February 2019. For a detailed timeline, see Additional file 1.

The final sample (*n* = 65) included 34 adolescents (13 female, 21 male; *m* = 14.56 years, *sd* = 1.46; range = 12–17) and 31 young adults (15 female, 16 male; *m* = 22.45 years, *sd* = 2.32; range = 18–27), resulting in an overall mean age of 18.32 years (*sd* = 4.41) at baseline.

### Ambulatory assessment

Participants took part in one week of ambulatory assessment in each of the three consecutive years. During these weeks, self-report data were collected via smartphone-based e-diaries (Motorola Moto G, Motorola Mobility LLC, Libertyville, Illinois, USA, www.motorola.com), while PA was continuously monitored using accelerometers (Move-II or Move-III, movisens GmbH, Germany, www.movisens.com).

E-diaries were triggered using a mixed sampling strategy that combined time-based and location-based triggers. Location-based triggers were initiated whenever a distance of more than 500 m was covered based on continuous geolocation monitoring to ensure broader spatial coverage of assessments and to capture greater within-person variability [29, 30]. Triggers occurred between 7:30 AM and 10:30 PM, with a minimum interval of 40 min and a maximum interval of 100 min between prompts; for participants attending school, triggers started only after school hours. This mixed sampling strategy resulted in approximately 10 to 23 possible e-diary prompts per day. The software movisensXS (version 0.6.3658) was used to implement the e-diaries and sampling protocol (movisens GmbH, Germany; https://xs.movisens.com).

### Measures

#### Mood

Mood was conceptualized as a three-dimensional construct comprising energetic arousal, valence, and calmness. It was assessed using two well-established e-diary questionnaires, tailored for adults and adolescents, respectively. In adults, mood was assessed using the six-item short scale of the Multidimensional Mood Questionnaire (MDMQ) [31] specifically developed to capture within-person mood dynamics [32]. Each dimension was measured with two bipolar items presented on visual analog scales ranging from 0 to 100: tired–awake and full of energy–without energy (energetic arousal), content–discontent and unwell–well (valence), relaxed–tense and agitated–calm (calmness). The MDMQ short scale is one of the most widely used and well-validated mood assessment instruments in EMA research [33].

In adolescents, mood was assessed using a 20-item unipolar scale specifically adapted for e-diary use with children and adolescents [34]. Items were rated on a 7-point Likert scale. Energetic arousal was measured with six items (active, concentrated, interested, exhausted, faint, tired), valence with nine items (cheerful, content, good, delighted, fantastic, afraid, mad, unhappy, miserable), and calmness with five items (pleasant, rested, anxious, on edge, stressed). The mood scale from Leonhard et al. [34] is the only validated EMA instrument to reliably assess mood within subjects in this age cohort.

All reverse-coded items were recoded so that higher values consistently indicated higher levels of the respective mood dimension. Mean scores were computed by averaging across the items for energetic arousal, valence, and calmness. Adolescent scores were linear transformed from the initial 1–7 scale to a 0–100 scale so that the lowest possible score corresponds to 0 and the highest to 100.

#### Physical activity

The Move-II and Move-III activity trackers, which contain a triaxial sensor recording acceleration at a sampling frequency of 64 Hz within a range of ± 8 g, were worn on the right side of the hip during waking hours throughout each seven-day assessment period. PA was operationalized as the mean Movement Acceleration Intensity (MAI) within the 60-minute interval preceding each e-diary assessment. MAI reflects the vector magnitude of the triaxial acceleration signal, high-pass filtered at 0.25 Hz to remove gravitational components and low-pass filtered at 11 Hz to eliminate artifacts [35]. MAI is expressed in units of standard gravity (g) and, in the present study, reported in deci-g (one-tenth of g) to improve interpretability and scaling.

### Analysis

#### Descriptives

We summarized physical activity (MAI in the 60-min interval prior to each e-diary) and all mood outcomes (energetic arousal, valence, calmness) using descriptive statistics (e.g., mean/SD and quartiles) and visualized their distributions across assessment weeks. For physical activity, we additionally examined diurnal variation by visualizing MAI across time of day. To quantify the proportion of variance in each mood outcome attributable to between-person differences, between–assessment-week differences within persons, and within–assessment-week fluctuations, we fitted unconditional three-level random-intercept models (assessments nested within assessment week nested within participants) and reported variance partition coefficients (VPCs).

#### Main analysis

We conducted three separate multilevel models with energetic arousal, valence, and calmness as outcomes, using PA aggregated in the 60 min prior to the e-diaries as the main predictor. This time window was selected based on previous research indicating that 60-minute aggregation periods yield reliable estimates of immediate associations between PA and psychological outcomes in everyday life [36]. Aggregating PA immediately before the EMA allowed us to specifically examine its momentary associations with mood.

Each model included the predictors “season” (four levels) to account for seasonal effects and both “time of day” (in hours relative to 7:30 a.m.) and its squared term to control for time-of-day effects. Both time variables were centered to improve model convergence and scaling. Where significant main effects of season emerged, we conducted Tukey-adjusted pairwise comparisons of estimated marginal means to further characterize differences between seasons. In addition, to examine whether the within-person PA–mood association differed across seasons, we conducted supplementary models including season × PA interaction terms.

To specifically model the within-subject associations of PA and mood, we person-mean centered the aggregated activity data separately for each of the three assessment weeks. An interaction between activity and assessment week was included to estimate week-specific activity effects. We additionally included sex and participant mean activity per assessment week as a control variables.

Although the data can in principle be conceptualized as having a three-level structure, assessment week was modeled as a within-person factor rather than as a separate level because our primary aim was to test whether the within-person PA–mood association differed across assessment weeks.

All models included a random intercept and a random interaction effect between activity and assessment week, allowing the activity–mood relationship to vary by participant and assessment week. Equation 1 presents the multilevel model specification for valence as the outcome; identical models were estimated separately for energetic arousal and calmness.

**Eq. 1**.

*Multilevel model: PA on valence*

1 $$\begin{aligned}\:valenc{e}_{\left\{ij\right\}}&=\:{\beta\:}_{0}+\:{\beta\:}_{1}\cdot\:seaso{n}_{\left\{ij\right\}}+\:{\beta\:}_{2}\cdot\:tim{e}_{\left\{ij\right\}}+\:{\beta\:}_{3}\cdot\:tim{e}_{\left\{ij\right\}}^{2}\\&+{\beta\:}_{4}\cdot\:{personmean\:activity}_{\left\{kj\right\}}+{\beta\:}_{5}\cdot\:sex+{\beta\:}_{6}\cdot\:activit{y}_{\left\{ij\right\}}\:\\&+\:{{\beta\:}_{7}\:\cdot\:year}_{\left\{kj\right\}}+\:{\beta\:}_{8}\:\cdot\:activit{y}_{\left\{ij\right\}}\cdot\:{year}_{\left\{kj\right\}}+\:{u}_{\left\{0j\right\}}\\&+\:{u}_{\left\{kj\right\}}\cdot\:\left(activit{y}_{\left\{ij\right\}}\cdot\:{year}_{\left\{kj\right\}}\right)+\:{\epsilon\:}_{\left\{ij\right\}}\end{aligned}$$.

Note. Effects are modeled on level 2 (subscript j = participant) and level 1 (subscript i = e-diary assessment); personmean activity = average activity per participant per assessment week (varies between assessment weeks but constant within assessment week); year = assessment week of the three consecutive years (k = 1,2,3); β_0_–β_7_ = fixed effects; u₀ⱼ = random intercept per participant; u_k_ⱼ = random effect per participant and year; ε_i_ⱼ = residual.

For each of the three models, we investigated the random effects (i.e., the random slopes) of PA on mood across participants and assessment weeks. Specifically, we examined the variance of the random slopes to quantify between-person differences as well as the correlations of the random slopes across the three weeks to assess the within-person stability of effects.

In order to make the random effects more interpretable and meaningful, we added the fixed main effect of PA and the fixed interaction of the respective assessment year to the random effects for each participant and assessment week. For each mood dimension, this yielded three participant-specific slopes per individual (one for each consecutive year), reflecting the individual effect of PA on mood rather than relative deviations from the group effect. We then modeled the individual PA-mood effects (i.e., the random slopes from the first series of multilevel models) in three separate random intercept models (energetic arousal, valence, and calmness) to compute the ICC of the individual effects (see Eq. 2).

**Eq. 2**.

*Random intercept model of the individual effects*

2 $$\:{slope}_{\left\{i\right\}}=\:{\beta\:}_{0}+\:{u}_{\left\{0i\right\}}+\:{\epsilon\:}_{\left\{i\right\}}$$.

The ICC indicates how much variance of the individual effects is due to between-person differences versus year-to-year variability. Thus, a high ICC means that most of the variability of the individual effects is due to consistent between-person differences, suggesting that individual effects are rather stable across the three yearly assessment weeks. Conversely, a low ICC indicates that the variability is largely due to fluctuations within participants across the three different weeks, implying less stability over time.

To explore potential sources of between-person variability in the within-person PA–mood associations, we conducted supplementary analyses focusing on sex and age group. First, extracted participant-specific PA–mood slopes were compared between groups using Welch’s t-tests. Second, sex and age group were each tested as moderators of the within-person PA–mood association in separate supplementary models including the respective interaction term with person-mean centered PA.

#### Sensitivity analysis

As previous research has shown that non-exercise activity (NEA; e.g. walking, gardening, or cleaning) and exercise can yield differential effects on energetic arousal, valence, and calmness [14, 16, 27, 37], we conducted an additional exploratory sensitivity analysis. For this purpose, we re-parameterized the main predictor, PA. Instead of aggregating all accelerometer data over the full 60 min preceding each e-diary, we separated light-to-moderate physical activity (LMPA) from vigorous physical activity (VPA). We used LMPA as a pragmatic proxy for NEA because many common non-exercise activities of daily living (e.g., walking, household tasks, gardening, or stair climbing) fall within the light-to-moderate intensity range, with several such activities classified above 3 METs in the Compendium of Physical Activities [38]. Based on established cutpoints for the hip-worn movisens Move accelerometer and the MAI metric used in this study [39], VPA was defined using a cutoff of 5.49 deci-g, and minute-level MAI values below this threshold were considered LMPA. To derive the LMPA-based predictor, minutes classified as VPA were set to NA prior to aggregation, so that higher values primarily reflected more light-to-moderate activity during the 60 min preceding the e-diary. Accordingly, aggregated accelerometer data within the LMPA range served as the main predictor and were person-mean centered within each assessment week. To control for the effect of VPA, the number of VPA minutes within the 60-minute period was included as a covariate in the model (see Eq. 3).

**Eq. 3**.

*Multilevel model: LMPA on valence*

3 $$\begin{aligned}\:valenc{e}_{\left\{ij\right\}}&=\:{\beta\:}_{0}+\:{\beta\:}_{1}\cdot\:seaso{n}_{\left\{ij\right\}}+\:{\beta\:}_{2}\cdot\:tim{e}_{\left\{ij\right\}}\\&+\:{\beta\:}_{3}\cdot\:tim{e}_{\left\{ij\right\}}^{2}+{\beta\:}_{4}\cdot\:{personmean\:activity}_{\left\{kj\right\}}\\&+{\beta\:}_{5}\cdot\:sex+\:{\beta\:}_{6}\cdot\:{VPA\:minutes}_{\left\{ij\right\}}+\:{\beta\:}_{7}\:\cdot\:{LMPA}_{\left\{ij\right\}}\\&+\:{{\beta\:}_{8}\:\cdot\:year}_{\left\{kj\right\}}+\:{\beta\:}_{9}\:\cdot\:{LMPA}_{\left\{ij\right\}}\cdot\:{year}_{\left\{kj\right\}}\\&+\:{u}_{\left\{0j\right\}}+\:{u}_{\left\{kj\right\}}\cdot\:\left({LMPA}_{\left\{ij\right\}}\cdot\:{year}_{\left\{kj\right\}}\right)+\:{\epsilon\:}_{\left\{ij\right\}}\end{aligned}$$.

Note. Effects are modeled on level 2 (subscript j = participant) and level 1 (subscript i = e-diary assessment); personmean activity = average activity per participant per assessment week (varies between assessment weeks but constant within assessment week); VPA minutes = number of vigorous PA minutes within the 60-minute period; LMPA = light-to-moderate PA; year = assessment week of the three consecutive years (k = 1,2,3); β_0_–β_7_ = fixed effects; u₀ⱼ = random intercept per participant; u_k_ⱼ = random effect per participant and year; ε_i_ⱼ = residual.

## Results

### Descriptives

#### Physical activity

**Fig. 1:**
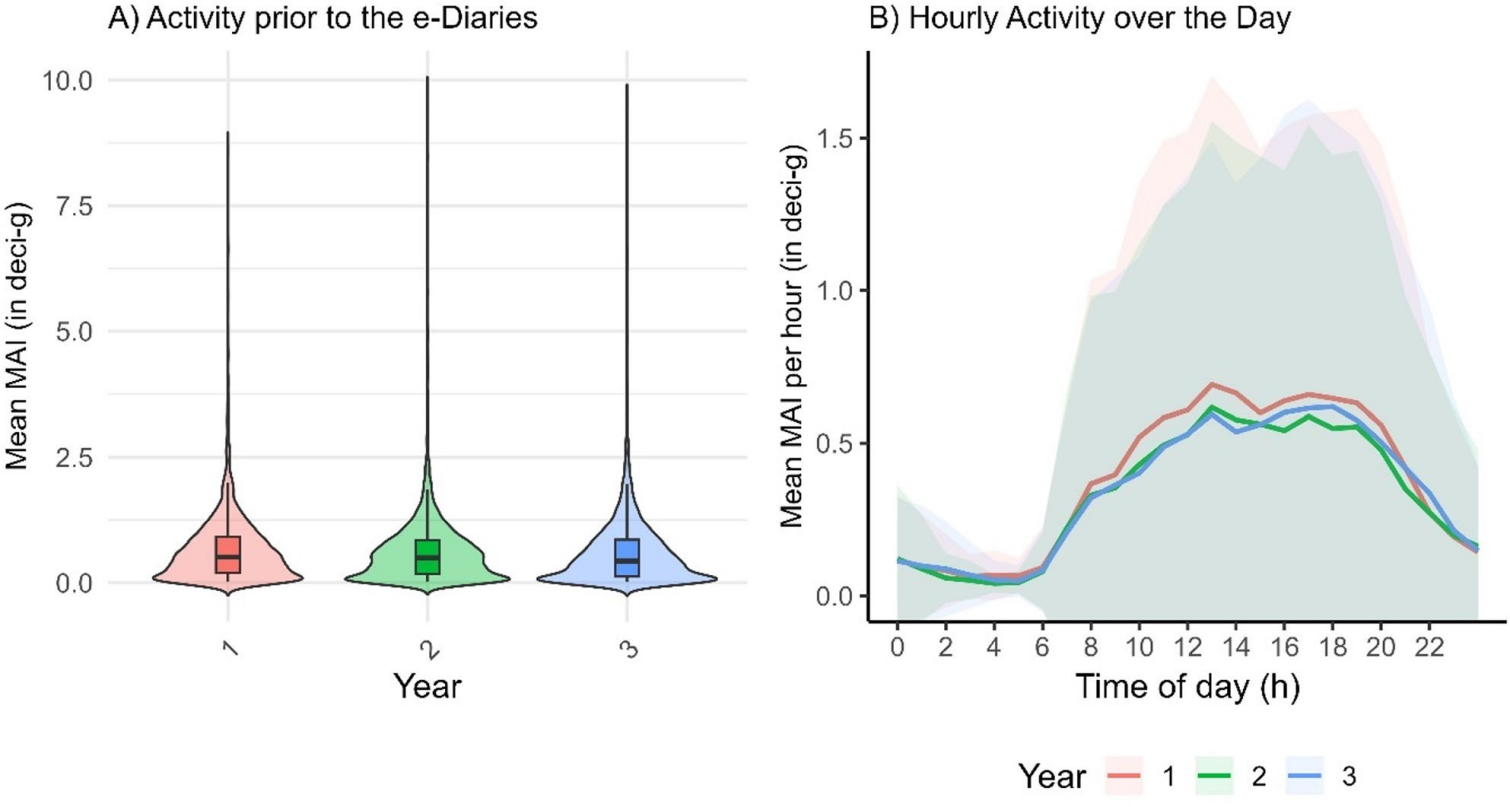
PA distribution across the three years (**A**) and over the day (**B**)

Participants were more often inactive than active in the 60-minute periods before the e-diary assessments. The mean MAI prior to e-diary entries was 0.63. To put it into context, MAI values recorded at the hip correspond approximately to tidying up at 1 deci-g, walking (3.2 km/h) at 2.5 deci-g, and running (7.6 km/h) at 8.8 deci-g [39]. The distribution of activity across the three years is shown in Fig. 1A. Participants were most active in the afternoon and evening. The distribution of hourly mean MAI scores across the day, separated by year, is depicted in Fig. 1B.

#### Mood

Overall, 10,913 e-diaries were completed during the three assessment weeks, corresponding to an average of 56 e-diaries per person per week or 7.99 per day. Compliance was high (*m* = 0.83, *sd* = 0.15).

**Fig. 2:**
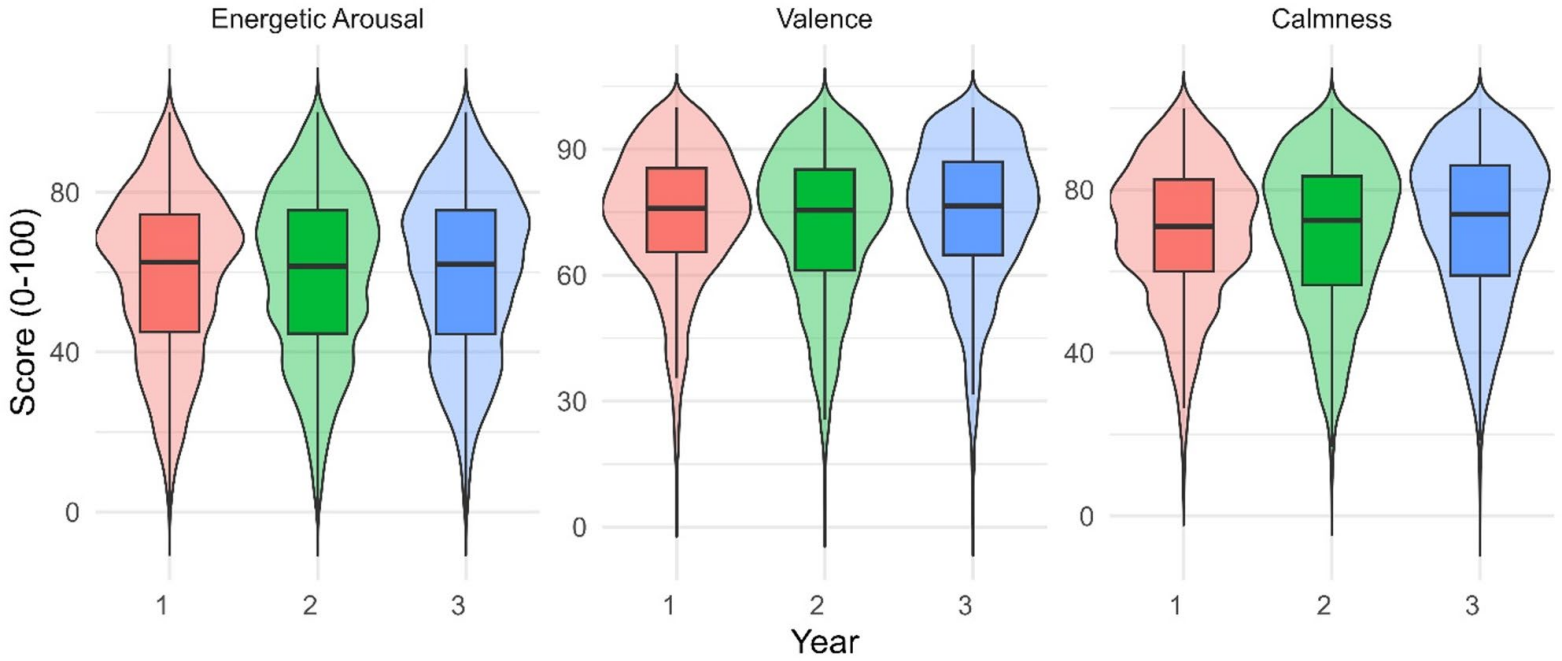
Mood distribution

Across all assessments, participants’ mean mood scores were 59.65 (*sd* = 20.64) for energetic arousal, 73.57 (*sd* = 16.92) for valence, and 70.12 (*sd* = 18.00) for calmness. The distribution of mood scores by year is shown in Fig. 2. For detailed summary statistics, see Table 1.

**Table 1:** Summary statistics of main predictor and outcome variables

	Min	Q1	Median	Mean (SD)	Q3	Max
mean MAI prior to e-diaries	0.03	0.17	0.48	0.63 (0.7)	0.87	9.8
energetic arousal	0	44.5	62	59.65 (20.64)	75	100
valence	1.85	64	75.93	73.57 (16.92)	86	100
calmness	0	59	73	70.12 (18)	83.5	100
compliance (per person per week)	0.3	0.73	0.87	0.83 (0.15)	0.94	1

#### Variance decomposition of mood outcomes

To describe how variability in mood was distributed across the three levels of the data structure, we derived VPCs from unconditional three-level random-intercept models. Most variability in mood was attributable to within–assessment-week fluctuations (energetic arousal: 0.73; valence: 0.65; calmness: 0.64). Between-person differences accounted for 0.21 (energetic arousal), 0.24 (valence), and 0.26 (calmness) of the total variance in each mood outcome, whereas between–assessment-week differences within persons accounted for 0.06, 0.11, and 0.10, respectively. Thus, across outcomes, 21–26% of variance reflected stable between-person differences, 6–11% reflected differences between assessment weeks within persons, and 64–73% reflected within–assessment-week variation. These estimates describe variance in the mood outcomes only and are independent of the physical activity–mood association analyses reported below.

### Main analysis

#### Fixed effects

As hypothesized, PA was significantly positively associated with subsequent energetic arousal and valence and significantly negatively associated with calmness. The strongest association was observed for energetic arousal (β = 4.88, *p* < .001; see Table 2), indicating that an increase in activity by 1 deci-g within the 60 min preceding the e-diary prompts was associated with a 4.88-point increase in energetic arousal on a 0–100 scale. It should be noted that the main-effect betas for activity refer to the reference year (year 3). In the absence of a significant interaction with year, the main-effect beta averaged across the three years was 4.79.

**Table 2:** Multilevel results for energetic arousal

Predictors	Estimates	std. Error	CI	df	t	*p*
Intercept	60.57	1.88	56.84–64.30	109.05	32.18	**< 0.001**
season [Spring]	3.42	0.93	1.59–5.24	7704.73	3.67	**< 0.001**
season [Summer]	4.06	0.82	2.45–5.67	8518.70	4.93	**< 0.001**
season [Winter]	1.61	0.78	0.08–3.15	8788.25	2.06	**0.039**
time centered	0.55	0.04	-0.63 – -0.47	10659.18	-13.07	**< 0.001**
time squared centered	-0.22	0.01	-0.24 – -0.20	10556.93	-23.56	**< 0.001**
mean PA per person per burst	1.71	1.28	-0.80–4.22	9059.62	1.33	0.183
sex [female]	0.59	2.47	-4.35–5.53	63.93	0.24	0.812
year [1]	-1.60	0.46	-2.50 – -0.69	10536.68	-3.46	**0.001**
year [2]	-0.85	0.42	-1.68 – -0.02	10514.87	-2.01	**0.045**
PA centered	4.88	0.71	3.43–6.32	39.13	6.84	**< 0.001**
year [1] × PA centered	-0.73	0.83	-2.41–0.95	38.41	-0.88	0.384
year [2] × PA centered	0.46	0.97	-1.51–2.43	38.12	0.47	0.638

Bold values indicate statistically significant effects at *p* < .05

PA was associated with a 1.27-point increase in valence for reference year 3 (see Table 3). In the absence of a significant interaction with year, the PA main-effect beta averaged across the three years was 1.58.

**Table 3:** Multilevel results for valence

Predictors	Estimates	std. Error	CI	df	t	*p*
intercept	74.35	1.69	71.00–77.71	100.58	43.98	**< 0.001**
season [Spring]	3.61	0.76	2.11–5.11	8575.47	4.73	**< 0.001**
season [Summer]	1.97	0.68	0.64–3.29	9258.73	2.91	**0.004**
season [Winter]	3.52	0.64	2.26–4.78	9401.39	5.48	**< 0.001**
time centered	0.25	0.03	0.18–0.32	10564.17	7.28	**< 0.001**
time squared centered	-0.01	0.01	-0.03–0.00	10451.38	-1.39	0.166
mean PA per person per burst	-2.81	1.05	-4.87 – -0.75	9629.39	-2.67	**0.008**
sex [female]	0.44	2.25	-4.06–4.94	62.63	0.20	0.845
year [1]	-0.44	0.38	-1.18–0.30	10546.48	-1.18	0.240
year [2]	-2.37	0.35	-3.05 – -1.69	10513.88	-6.83	**< 0.001**
PA centered	1.27	0.61	0.05–2.48	47.09	2.09	**0.042**
year [1] × PA centered	0.14	0.71	-1.30–1.59	35.21	0.20	0.840
year [2] × PA centered	0.77	0.78	-0.81–2.34	41.54	0.99	0.330

Bold values indicate statistically significant effects at *p* < .05

Whereas PA was positively related to higher energetic arousal and valence, it was negatively associated with calmness. Meaning, in reference year 3, calmness decreased by 1.86 points following an increase of 1 deci-g in activity within the preceding 60 min (see Table 4). Again, with no significant interaction with year, the average PA main-effect beta across the three years was − 1.67.

**Table 4:** Multilevel results for calmness

Predictors	Estimates	std. Error	CI	df	t	*p*
intercept	69.36	1.85	65.70–73.03	95.15	37.56	**< 0.001**
season [Spring]	2.46	0.81	0.87–4.05	8778.73	3.03	**0.002**
season [Summer]	2.60	0.72	1.19–4.00	9367.38	3.61	**< 0.001**
season [Winter]	5.47	0.68	4.13–6.81	9540.45	8.00	**< 0.001**
time centered	0.23	0.04	0.16–0.30	10512.94	6.29	**< 0.001**
time squared centered	0.03	0.01	0.01–0.04	9138.03	3.35	**0.001**
mean PA per person per burst	-2.92	1.12	-5.11 – -0.73	9447.48	-2.62	**0.009**
sex [female]	0.56	2.53	-4.49–5.62	63.73	0.22	0.825
year [1]	-1.81	0.40	-2.60 – -1.03	10082.93	-4.53	**< 0.001**
year [2]	-1.84	0.37	-2.56 – -1.12	10028.30	-5.00	**< 0.001**
PA centered	-1.86	0.53	-3.05 – -0.67	9.53	-3.51	**0.006**
year [1] × PA centered	0.36	0.67	-1.03–1.76	21.76	0.54	0.594
year [2] × PA centered	0.20	0.59	-15.13–15.53	0.77	0.34	0.803

Bold values indicate statistically significant effects at *p* < .05

Regarding the covariates, mean PA per person per burst was negatively associated with valence (β = -2.81, *p* = .008) and calmness (β = -2.92, *p* = .009), indicating that participants with higher average PA levels within a given burst tended to report lower levels of valence and calmness. Season (reference level: autumn) also showed significant associations with energetic arousal, valence, and calmness. To further clarify seasonal effects, we conducted Tukey-adjusted pairwise comparisons of estimated marginal means. For energetic arousal, levels were lower in autumn than in spring (mean difference = 3.42, *p* = .001) and summer (mean difference = 4.06, *p* < .001), and lower in winter than in summer (mean difference = 2.45, *p* = .028). For valence, levels were lower in autumn than in spring (mean difference = 3.61, *p* < .001), summer (mean difference = 1.97, *p* = .019), and winter (mean difference = 3.52, *p* < .001), and higher in spring than in summer (mean difference = 1.64, *p* = .029). For calmness, levels were higher in winter than in spring (mean difference = 3.01, *p* < .001), summer (mean difference = 2.87, *p* = .001), and autumn (mean difference = 5.47, *p* < .001), and lower in autumn than in spring (mean difference = 2.46, *p* = .013) and summer (mean difference = 2.60, *p* = .002). Taken together, these follow-up contrasts suggest that autumn was consistently associated with lower mood, whereas winter was associated with particularly high calmness. To further examine seasonal differences, we tested season as a moderator of the within-person PA–mood association, but no significant interaction effects emerged for energetic arousal, valence, or calmness (see Additional File 2).

#### Within and between person variability of individual effects

The random effects of PA on mood also showed considerable variability. For energetic arousal, the variance of the random effects ranged from 12.55 to 21.45 across the three years; for valence, it ranged from 1 to 18.81; and for calmness, from 6.77 to 11.25, indicating notable between-person differences in the associations of PA and mood (see Table 5).

**Table 5:** Random effects of the three multilevel models

		energetic arousal			valence			calmness		
Variance	Residual	293.44			195.98			220.64		
	Intercept	100.08			86.60			100.50		
	PA:year3	14.37			11.04			6.76		
	PA:year1	12.55			1.00			10.41		
	PA:year2	21.45			18.81			11.25		
Correlations		Intercept	PA:year3	PA:year1	Intercept	PA:year3	PA:year1	Intercept	PA:year3	PA:year1
	PA:year3	-0.22			-0.19			0.09		
	PA:year1	-0.05	0.62		-0.64	0.13		-0.12	0.68	
	PA:year2	-0.30	0.40	0.53	-0.32	0.58	0.39	0.13	0.96	0.48
ICC		0.26			0.31			0.32		
N _Participant_		65			65			65		
Observations		10743			10743			10742		
Marginal R^2^ / Conditional R^2^		0.079 / 0.322			0.022 / 0.328			0.022 / 0.337		

Moreover, small-to-moderate negative correlations between the random slopes and the random intercepts for energetic arousal (-0.05 to -0.30) and valence (-0.19 to -0.64) indicate that the associations of PA and energetic arousal as well as valence was stronger among participants whose average levels of these mood dimensions were lower. For calmness, the correlations between the random intercepts and the random slopes of PA were mixed (see Table 5).

As hypothesized, we found small-to-high correlations of the random slopes across the three assessment weeks. For energetic arousal, correlations ranged from 0.40 to 0.62; for valence, from 0.13 to 0.58; and for calmness, from 0.48 to 0.96. These correlations indicate that within-subject effects show some degree of person-specific consistency over three consecutive years, meaning that those with stronger associations in one year also show stronger associations in the other years, and vice versa.

**Fig. 3:**
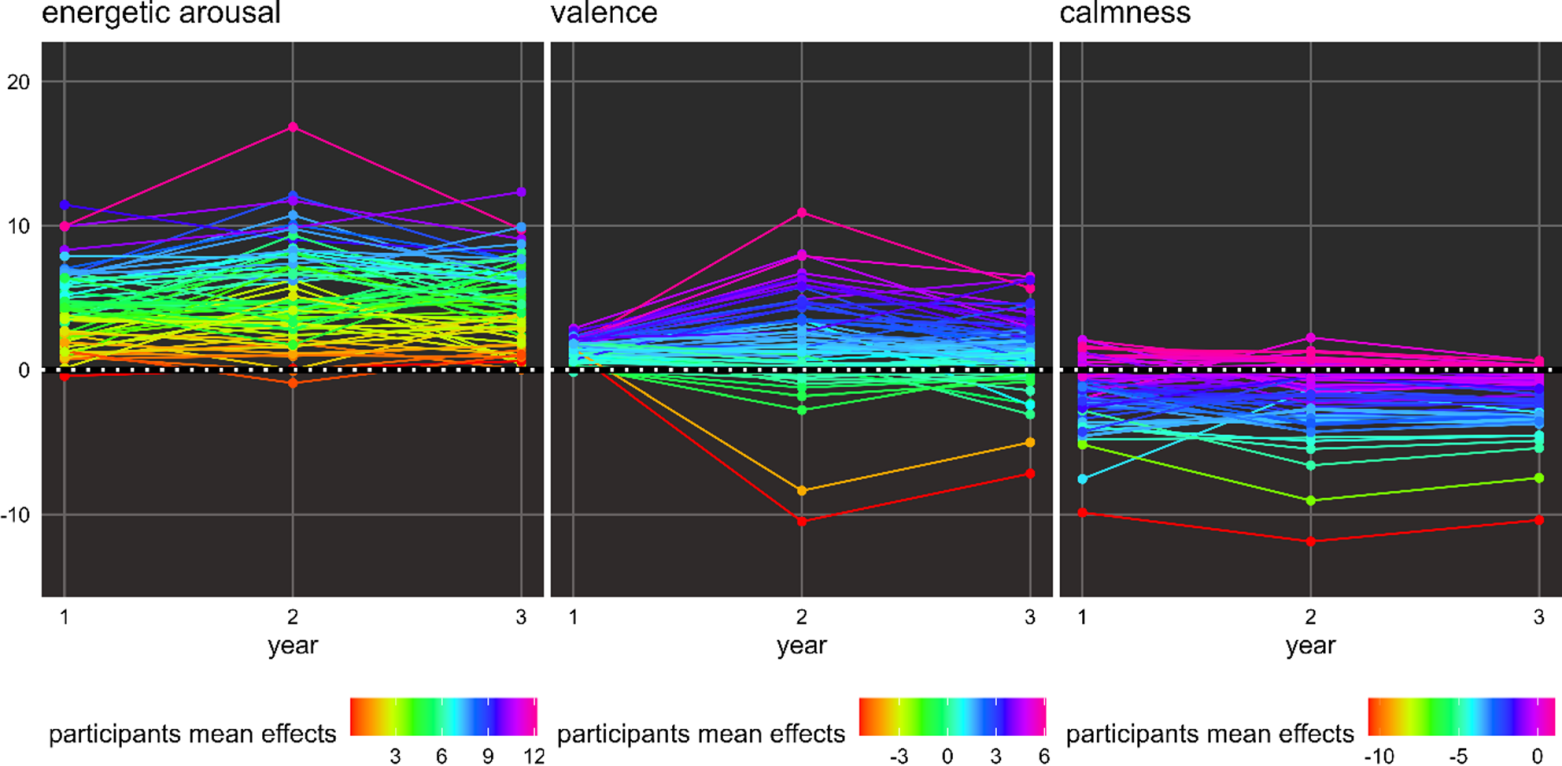
Individual effects across three years

To obtain interpretable individual effects, we added the fixed main effect of PA and the corresponding fixed interaction to the random slopes (see Fig. 3). In Fig. 3, each of the three points on a line represents the individual effects for a participant at one of the three years. Although individual effects show some fluctuations over time, consistent differences between participants are evident across the three years.

To assess the relative between- and within-subject variance of the participant-specific effects per year, we calculated the ICC by fitting a random intercept model for each mood dimension using the individual slopes.

The ICC for PA-energetic arousal slopes was 0.64, indicating that 64% of the variance in individual associations of PA and energetic arousal is attributable to between-subject differences (i.e., trait-like variability). The remaining 36% of the variance reflects within-subject fluctuations across the three years (i.e., state-like variability). For valence, the ICC was 0.441, and for calmness it was 0.797, reflecting measurable and relatively stable between-subject differences of the associations of PA and all three mood dimensions.

**Fig. 4:**
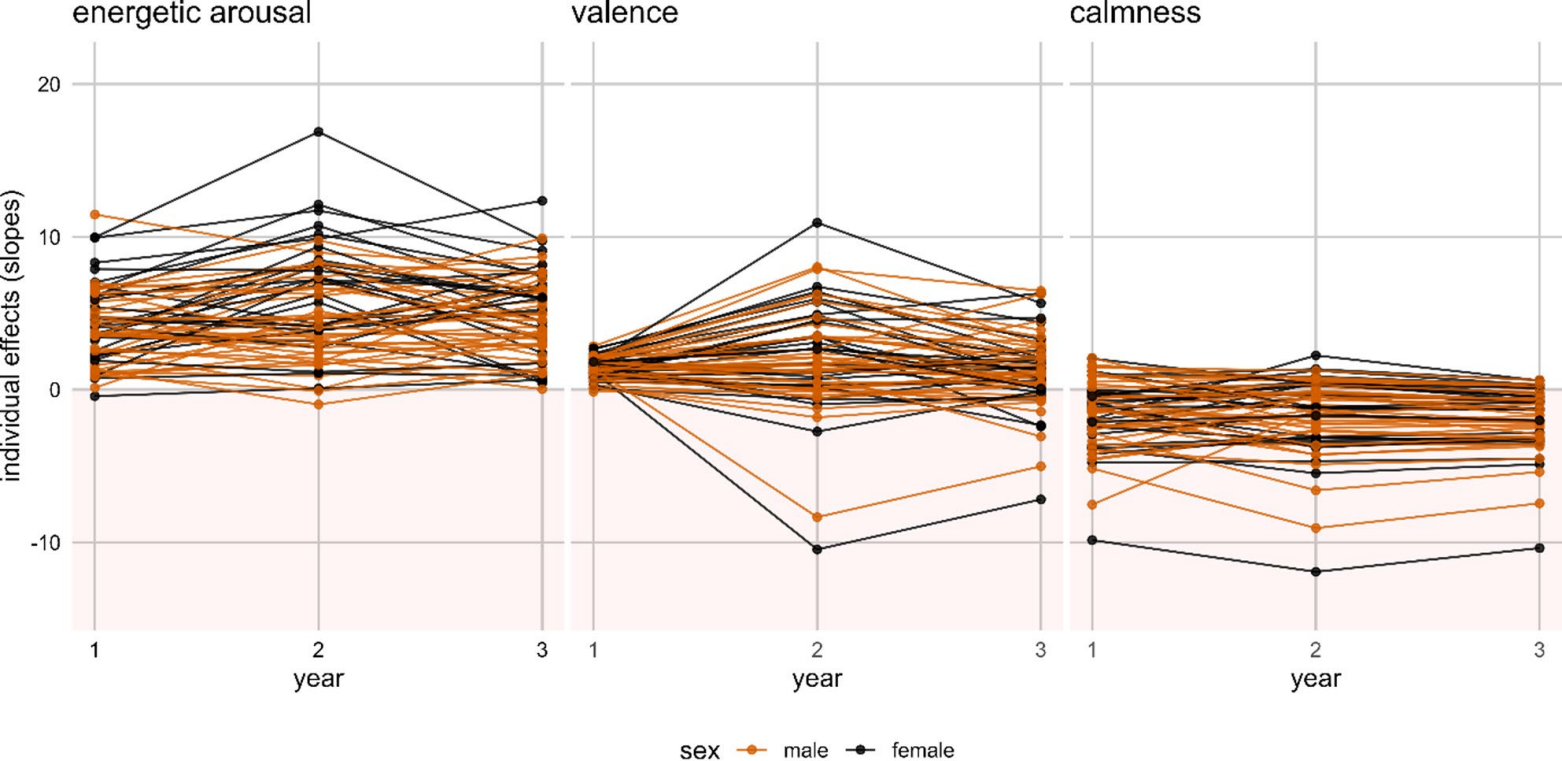
Individual effects across three years by sex

Building on this evidence for substantial between-person variability in the within-person PA–mood associations, we explored whether these individual differences were related to sex and age. In exploratory comparisons of the extracted participant-specific slopes, we conducted Welch’s t-tests to examine differences by sex and age group. No statistically significant differences emerged by sex across any mood dimension (Fig. 4; Table 6). In addition, we formally tested sex as a potential moderator by including sex × PA interaction terms in three-level multilevel models (assessments nested within assessment weeks nested within participants) and found no evidence that within-person PA–mood associations were moderated by sex. Results of these interaction models are provided in Additional File 2.

**Table 6:** Participant-specific PA–mood slopes by sex

	male m (sd)	female m (sd)	t (df)	*p*
energetic arousal	4.23 (2.23)	5.52 (2.82)	1.99 (df = 50.1)	0.052
valence	1.53 (1.78)	1.62 (2.13)	-0.17 (df = 52.1)	0.867
calmness	-1.66 (1.87)	-1.7 (2.40)	0.07 (df = 49.7)	0.942

**Fig. 5:**
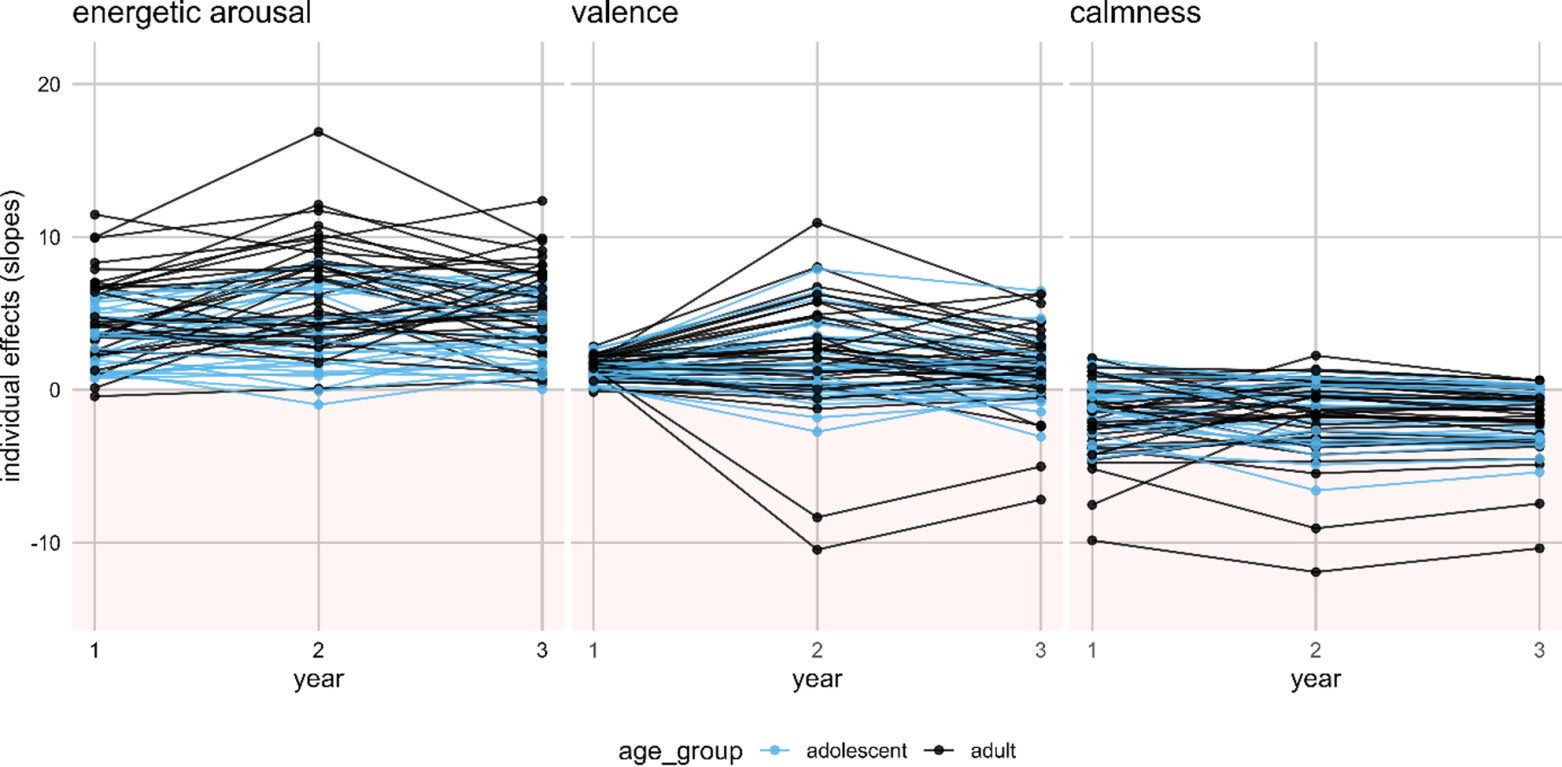
Individual effects across three years by age group

Likewise, no age-group differences were found in the individual PA–valence or PA–calmness slopes. However, for energetic arousal, the extracted participant-specific slopes differed significantly between adolescents and adults (Fig. 5; Table 7).

**Table 7:** Participant-specific PA–mood slopes by age group

	adolescents m (sd)	adults m (sd)	t (df)	*p*
energetic arousal	3.65 (2.10)	5.60 (2.58)	3.35 (df = 61.8)	< 0.001
valence	1.39 (1.55)	1.70 (2.16)	-0.66 (df = 63.0)	0.512
calmness	-1.48 (1.63)	-1.81 (2.38)	0.67 (df = 62.9)	0.508

To test a potential moderating effect of age group on the within-person PA–mood associations, we estimated supplementary three-level interaction models (assessments nested within assessment weeks nested within participants) including age group × PA interaction terms. No significant age-group differences were found in the within-person PA effects on valence or calmness, whereas the within-person PA effect on energetic arousal was significantly moderated by age group (β = 3.47, *p* < .001), indicating that adults showed a stronger increase in energetic arousal after physical activity compared to adolescents. Detailed results are reported in Additional File 2.

### Sensitivity analysis

In order to specifically examine the effects of everyday life activity on mood, we conducted an exploratory sensitivity analysis with light-to-moderate PA (LMPA) as predictor. The fixed effects were largely similar to those in the main analysis; however, they were only significant for the outcomes energetic arousal and calmness and nonsignificant for valence. Moreover, the effect estimates of LMPA on energetic arousal and calmness were slightly larger than those of overall PA in the main models: the LMPA main-effect betas averaged across the three years were β = 5.93 for energetic arousal (compared to β = 4.79 in the main model), and β = -1.82 for calmness (compared to β = -1.67 in the main model; see Additional File 2 for complete results tables).

The random effects of LMPA on mood generally showed higher variance compared to the main analysis (see Table 8). For energetic arousal, variance ranged from 26.13 to 30.76; for valence, from 6.45 to 26.03; and for calmness, from 15.70 to 19.84, indicating increased between-person differences in the effects of LMPA on mood compared to the main analysis.

**Table 8:** Random effects of the three multilevel models with LMPA as predictor

		energetic arousal			valence			calmness		
Variance	Residual	291.18			195.32			219.78		
	Intercept	99.71			86.57			101.54		
	PA:year3	26.13			16.84			15.70		
	PA:year1	30.76			6.45			16.06		
	PA:year2	28.86			26.03			19.84		
Correlations		Intercept	PA:year3	PA:year1	Intercept	PA:year3	PA:year1	Intercept	PA:year3	PA:year1
	PA:year3	-0.28			-0.18			0.08		
	PA:year1	-0.10	0.24		-0.37	-0.13		-0.13	0.4	
	PA:year2	-0.25	0.42	0.38	-0.31	0.46	0.38	0.13	0.4	0.28
ICC		0.26			0.31			0.32		
N _Participant_		65			65			65		
Observations		10743			10743			10742		
Marginal R^2^ / Conditional R^2^		0.075 / 0.319			0.021 / 0.328			0.022 / 0.337		

Correlations of the random effects across the three assessment weeks were generally smaller. For energetic arousal, correlations ranged from 0.24 to 0.42; for valence, from − 0.13 to 0.46; and for calmness, from − 0.13 to 0.40. This indicates a lower consistency of within-subject LMPA effects across the three assessment weeks compared to the main analysis.

The analysis of the individual effects revealed moderate within- and between-subject variability in the estimated LMPA effects on mood, with ICCs of 0.49 (energetic arousal), 0.37 (valence), and 0.51 (calmness), all smaller than in the main analysis. This indicates that the individual effects of LMPA on mood were less stable within participants across the three assessment weeks than the individual effects of general PA on mood in the main analysis.

## Discussion

Here, we show that momentary within-person associations between physical activity and mood in everyday life comprise a stable trait-like component, yet also fluctuate over time within individuals. This indicates that PA-mood associations are not fixed but partly shaped by putative modifiable state-like influences. Our intensive three-year longitudinal study, including one week of ambulatory assessment per year, revealed that PA was associated with increased subsequent energetic arousal and valence and decreased calmness. These effects varied both between individuals and within individuals across years, with the variance explained being moderately balanced across within- and between-person fluctuations, depending on the mood dimension and the type of PA considered.

Supporting our hypotheses, we found that PA was positively associated with subsequent energetic arousal and valence and negatively associated with calmness, which aligns with previous research [14, 40–43]. Of the three mood dimensions, energetic arousal exhibited the strongest association, which is consistent with prior studies showing that PA effects on energy are typically of the highest effect size compared to other mood dimensions [6].

Additional covariate findings also merit consideration. Mood levels differed across seasons, with autumn generally associated with lower mood and winter with particularly high calmness. Importantly, additional interaction analyses indicated that season did not moderate the within-person PA–mood association, suggesting that season influenced overall mood levels, but not the within-person association between PA and mood. Moreover, higher mean PA per person per burst was associated with lower valence and calmness. The opposing directions of the within-person and between-person associations highlight that these levels of analysis should not be interpreted interchangeably and underscore the importance of examining within-person associations at the within-person level [44]. Interpreting group-level data as if they reflected individual-level processes is referred to as the ecological fallacy [45]. Consistent with this, prior work has shown that PA–stress associations differ at the between-person and within-person levels and can even point in opposite directions [46].

Investigation of the random effects showed that between-person differences in the associations of PA and mood were observable in all three years. Further, they were correlated across the three assessments, suggesting that within-subject associations of PA and mood show some degree of person-specific consistency over time. ICCs of the individual associations of PA and mood further indicate that a substantial portion of the variance is attributable to stable between-person differences: 64% for energetic arousal, 44.1% for valence, and 79.7% for calmness, with the remaining variance reflecting within-person variability across the three years. These findings provide evidence that the individual associations of PA and mood are not purely situational but include a consistent, person-specific component. Thus, individuals differ in how PA associates with mood, and a portion of these differences remains relatively stable over years. In other words, some individuals may benefit more from PA than others, potentially due to person-specific factors that are relatively stable over time (e.g., biological factors, personal lifestyle), emphasizing the importance of tailoring PA interventions rather than relying on a one-size-fits-all approach.

One possible source of these between-person differences may be age-related variation. In our supplementary analyses, sex did not moderate the within-person PA–mood associations, whereas age-group differences emerged for energetic arousal, with adults showing a stronger positive association between PA and subsequent energetic arousal than adolescents. However, this finding should be interpreted cautiously, as age group and questionnaire version were not independent in our design. Accordingly, the observed difference may reflect age-related differences, differences in item composition, or a combination of both.

Notably, we found small-to-moderate negative correlations between the random effects and the intercepts for energetic arousal (-0.05 to -0.30) and valence (-0.19 to -0.64), which suggest that effects of PA on energetic arousal and valence were stronger among participants with lower average levels of these mood dimensions. This highlights the potential benefit of PA for individuals who typically feel less energetic or more downcast and is consistent with previous research showing that PA may be particularly effective in reducing depressive symptoms across both clinical and non-clinical populations [2, 47].

However, a substantial portion of the variability in effect sizes remains within-subject, which may reflect factors that change over weeks or years, such as situational context, life events, weather, or random measurement noise. For instance, previous research has demonstrated that weather conditions can moderate within-subject associations between affective states and physical behavior [17], and that positive PA-mood associations tend to be stronger when PA is performed outdoors compared to indoors [48]. Thus, effect sizes of PA and mood associations are shaped not only by stable individual characteristics but also by situational factors, suggesting that context-sensitive and adaptive approaches, such as just-in-time adaptive interventions (JITAIs), may be particularly effective in maximizing PA benefits.

Given that prior studies suggest the differential associations of PA and mood for exercise and NEA, we additionally conducted a sensitivity analysis focusing specifically on NEA, using LMPA as the main predictor. In contrast to our main analysis and consistent with previous research on NEA [16, 37], we found no significant associations of NEA with valence. Associations of NEA with energetic arousal and calmness were slightly larger compared to the associations with overall PA in the main analysis, which aligns with prior research showing that effects on these dimensions can primarily be observed for NEA. Compared to the main analysis, random slopes showed higher variance and correlations across the three assessment weeks were generally smaller in the sensitivity analysis. ICCs of the individual LMPA effects were also lower. Thus, within-subject associations of LMPA and mood were somewhat less stable across the three years than associations of mood with general PA. A possible explanation might be that, when specifically examining NEA, the effects are less stable because these activities are highly heterogeneous and context-dependent (e.g., a walk in the park versus shopping in a noisy supermarket). In contrast, we are tempted to speculate that effects of exercise on mood exhibit more stable effects within persons across time.

Future research could build on existing evidence of moderating effects to clarify which factors contribute to stable, trait-like components of PA associations with mood and which drive the variability in within-person reactivity over time. In particular, identifying modifiable components remains a key avenue for developing effective behavior-change interventions. AA studies would benefit from explicitly distinguishing within-person processes into stable (potentially heritable) and flexible, context-dependent components. This approach could enhance mechanistic understanding and guide the design of interventions tailored to both individual traits and situational responsiveness, emphasizing that capturing within-person associations alone may be insufficient to fully understand PA-mood dynamics.

While our study provides valuable insights into within- and between-person variability of PA-mood associations, there are some limitations that need to be considered. First, the investigation of individual differences in the associations of PA and subsequent mood is inherently sample-specific, potentially limiting the generalizability of our results. However, by using a carefully stratified community-based sample that reflects key population characteristics such as age, sex, and nationality, the impact of this limitation is likely limited. Second, to account for potential age-related differences in how mood is experienced and reported, we used age-appropriate mood assessment methods for adolescents and adults and harmonized the scales according to an established procedure in the EMA field [34]. Supplementary moderation analyses showed a significant difference between adolescents and adults in the within-person PA effect on energetic arousal, but no such difference emerged for valence or calmness. Because age group and questionnaire version were not independent in our design, this interaction cannot be interpreted as reflecting a pure age effect or a pure measurement effect. However, including both adolescents and young adults, spanning a broad age range characterized by ongoing emotional and physiological change, facilitated a particularly rigorous examination of the stability of PA-mood associations. Third, our study design was observational in nature. While real-life studies maximize ecological validity of findings, they are also limited regarding potential confounding variables. Although our temporal parameterization, linking PA immediately prior to mood assessments, allows us to demonstrate directional associations, no firm causal conclusions can be drawn from our design. To explicitly examine causal effects of PA on mood in daily life, designs such as within-person encouragement interventions are warranted in future studies [49].

## Conclusions

Our findings replicate previous evidence that PA is associated with subsequent mood. Notably, even light-to-moderate everyday life activities are substantially associated with enhanced feelings of energy, highlighting the relevance of everyday life movement for well-being. Our results demonstrate that short ambulatory assessment periods, such as one week with approximately eight e-diary prompts per day, are sufficient to capture person-specific within-subject associations of PA and mood.

For the first time, we show that the within-subject associations of PA and mood entail both stable trait and variable state components. While differences in the associations are partly attributable to person-specific factors, a substantial amount of within-subject variability exists. PA-mood associations are also partly context-dependent, and PA interventions may be more effective at certain times or under specific circumstances. This highlights the potential of personalized interventions building both upon individualized and context-dependent PA-mood phenotypes; for example, context-sensitive, adaptive interventions, such as JITAIs, which can deliver prompts to engage in PA at moments when they are likely to have the greatest impact.

Taken together, the findings suggest PA-mood associations reflect a combination of stable individual traits and within-person variability, highlighting the potential of personalized and context-sensitive strategies.

## Supplementary Information

Additional file 1.

Additional file 2.

## Data Availability

The datasets used and/or analyzed during the current study are available from the corresponding author on reasonable request.

## Abbreviations

PA: physical activity
AA: ambulatory assessment
EMA: ecological momentary assessment
ESM: experience sampling method
CIMH: Central Institute of Mental Health
MDMQ: Multidimensional Mood Questionnaire
MAI: Movement Acceleration Intensity
VPC: variance partition coefficient
ICC: intraclass correlation coefficient
NEA: non-exercise activity
LMPA: light-to-moderate physical activity
VPA: vigorous physical activity
JITAI: just-in-time adaptive intervention
