# Supplementary figures and images for: Physical activity and mood in daily life – a multi-burst ambulatory assessment study disentangling state and trait components of within-person associations
Source: Int J Behav Nutr Phys Act. 2026 May 14;23:70. doi: 10.1186/s12966-026-01932-x (PMC13343692; doi:10.1186/s12966-026-01932-x)

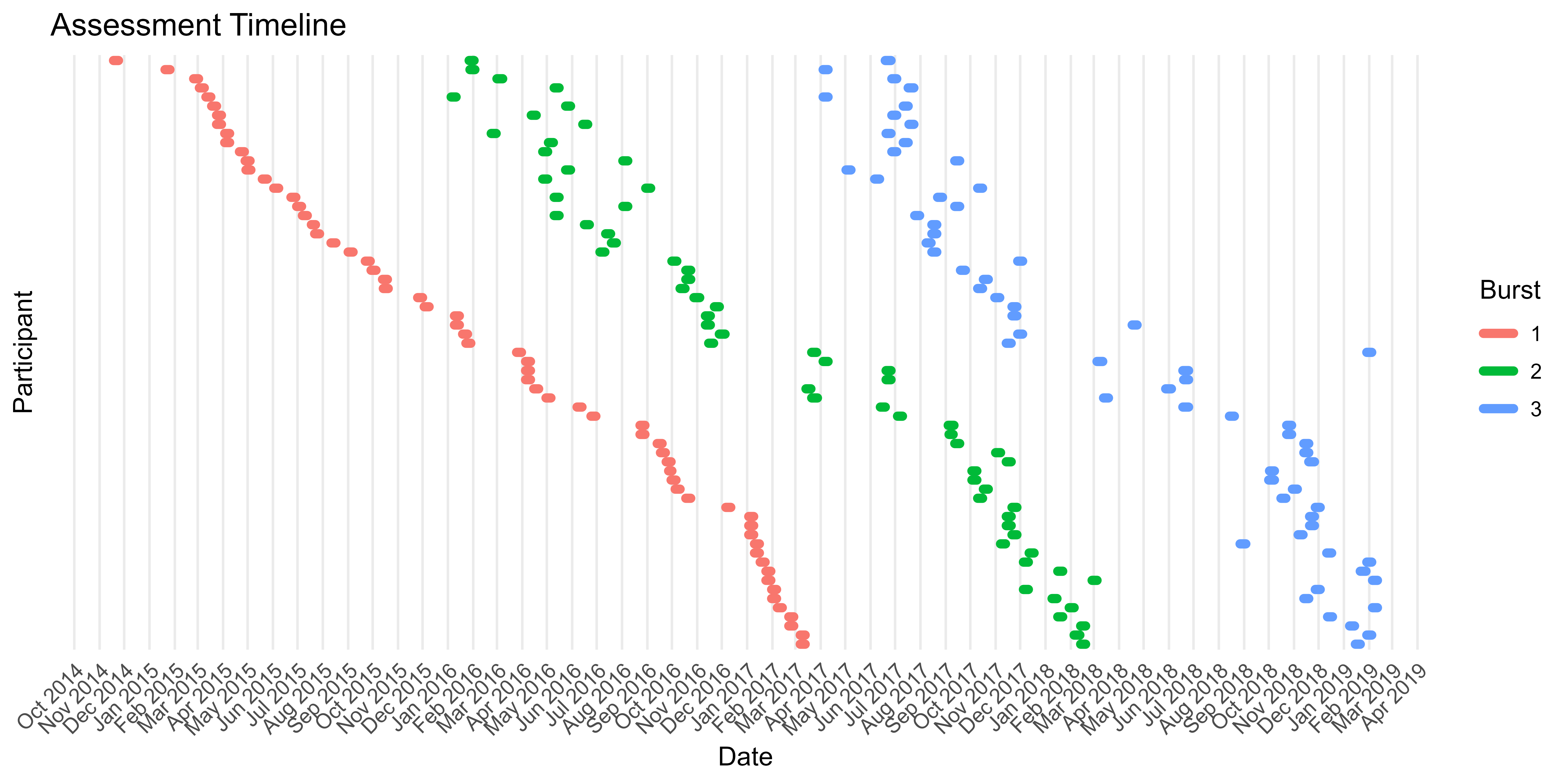

Supplement: Supplementary file 1 — Additional file 1. [file 12966_2026_1932_MOESM1_ESM.png]
