# Supplementary material for: Physical activity and mood in daily life – a multi-burst ambulatory assessment study disentangling state and trait components of within-person associations
Source: Int J Behav Nutr Phys Act. 2026 May 14;23:70. doi: 10.1186/s12966-026-01932-x (PMC13343692; doi:10.1186/s12966-026-01932-x)
Supplement: Supplementary file 2 — Additional file 2. [file 12966_2026_1932_MOESM2_ESM.docx]

**Supplementary Analyses**

[1 Moderation Analyses: Season 1](#_Toc224810149)

[1.1 Season × PA on Energetic Arousal 1](#_Toc224810150)

[1.2 Season × PA on Valence 2](#_Toc224810151)

[1.3 Season × PA on Calmness 3](#_Toc224810152)

[2 Moderation Analyses: Sex 4](#_Toc224810153)

[2.1 Sex × PA on Energetic Arousal 4](#_Toc224810154)

[2.2 Sex × PA on Valence 5](#_Toc224810155)

[2.3 Sex × PA on Calmness 6](#_Toc224810156)

[3 Moderation Analyses: Age group 7](#_Toc224810157)

[3.1 Age group × PA on Energetic Arousal 7](#_Toc224810158)

[3.2 Age group × PA on Valence 8](#_Toc224810159)

[3.3 Age group × PA on Calmness 9](#_Toc224810160)

# Moderation Analyses: Season

## Season × PA on Energetic Arousal

**Table 1***Multilevel results for Energetic Arousal*

| Predictors | Estimates | std. Error | CI | t | p | df |
| --- | --- | --- | --- | --- | --- | --- |
| (Intercept) | 61.66 | 2.60 | 56.53 – 66.79 | 23.70 | **<0.001** | 179.13 |
| time centered | -0.55 | 0.04 | -0.63 – -0.47 | -13.52 | **<0.001** | 10568.55 |
| time squared centered | -0.25 | 0.01 | -0.26 – -0.23 | -26.30 | **<0.001** | 10686.31 |
| mean PA per person per burst | 1.79 | 2.93 | -3.98 – 7.57 | 0.61 | 0.540 | 170.86 |
| sex [female] | -0.41 | 2.53 | -5.47 – 4.65 | -0.16 | 0.871 | 67.09 |
| year [1] | -1.90 | 1.13 | -4.13 – 0.33 | -1.68 | 0.095 | 135.02 |
| year [2] | -1.60 | 1.07 | -3.72 – 0.53 | -1.49 | 0.140 | 123.72 |
| season [Spring] | 5.23 | 1.81 | 1.67 – 8.79 | 2.89 | **0.004** | 352.99 |
| season [Summer] | 3.08 | 1.62 | -0.12 – 6.27 | 1.90 | 0.059 | 382.05 |
| season [Winter] | 1.18 | 1.33 | -1.44 – 3.80 | 0.89 | 0.375 | 711.19 |
| PA centered | 4.19 | 0.72 | 2.76 – 5.61 | 5.81 | **<0.001** | 131.36 |
| season [Spring] × PA centered | 1.05 | 0.99 | -0.90 – 3.00 | 1.06 | 0.291 | 373.72 |
| season [Summer] × PA centered | -0.29 | 0.90 | -2.06 – 1.47 | -0.33 | 0.744 | 631.93 |
| season [Winter] × PA centered | 0.62 | 1.01 | -1.37 – 2.61 | 0.61 | 0.542 | 346.49 |

**Table 2***Type III Analysis of Variance Table with Satterthwaite's method*

| Predictors | F | p | df |
| --- | --- | --- | --- |
| time centered | 182.73 | **<0.001** | 10568.55 |
| time squared centered | 691.51 | **<0.001** | 10686.31 |
| mean PA per person per burst | 0.38 | 0.540 | 170.86 |
| sex | 0.03 | 0.871 | 67.09 |
| year | 1.67 | 0.193 | 127.57 |
| season | 2.88 | **0.036** | 360.24 |
| PA centered | 76.92 | **<0.001** | 60.24 |
| season : PA centered | 0.72 | 0.540 | 447.20 |

## Season × PA on Valence

**Table 3***Multilevel results for Valence*

| Predictors | Estimates | std. Error | CI | t | p | df |
| --- | --- | --- | --- | --- | --- | --- |
| (Intercept) | 72.84 | 2.43 | 68.05 – 77.64 | 29.99 | **<0.001** | 180.57 |
| time centered | 0.25 | 0.03 | 0.19 – 0.32 | 7.72 | **<0.001** | 10559.87 |
| time squared centered | -0.02 | 0.01 | -0.03 – -0.00 | -2.42 | **0.016** | 10660.77 |
| mean PA per person per burst | 0.70 | 2.84 | -4.91 – 6.31 | 0.25 | 0.805 | 177.52 |
| sex [female] | -0.03 | 2.25 | -4.52 – 4.46 | -0.01 | 0.991 | 65.30 |
| year [1] | -0.77 | 1.11 | -2.98 – 1.43 | -0.70 | 0.488 | 134.28 |
| year [2] | -2.80 | 1.07 | -4.91 – -0.69 | -2.63 | **0.010** | 123.14 |
| season [Spring] | 4.72 | 1.65 | 1.47 – 7.96 | 2.86 | **0.004** | 441.36 |
| season [Summer] | 1.18 | 1.47 | -1.71 – 4.07 | 0.80 | 0.422 | 514.32 |
| season [Winter] | 2.92 | 1.17 | 0.63 – 5.21 | 2.50 | **0.013** | 1084.36 |
| PA centered | 1.79 | 0.56 | 0.67 – 2.91 | 3.18 | **0.002** | 91.12 |
| season [Spring] × PA centered | 0.06 | 0.78 | -1.48 – 1.60 | 0.08 | 0.937 | 227.55 |
| season [Summer] × PA centered | -1.40 | 0.71 | -2.80 – 0.00 | -1.96 | 0.051 | 390.12 |
| season [Winter] × PA centered | -0.37 | 0.80 | -1.94 – 1.20 | -0.46 | 0.644 | 204.07 |

**Table 4***Type III Analysis of Variance Table with Satterthwaite's method*

| Predictors | F | p | df |
| --- | --- | --- | --- |
| time centered | 59.64 | <0.001 | 10559.87 |
| time squared centered | 5.85 | 0.016 | 10660.77 |
| mean PA per person per burst | 0.06 | 0.805 | 177.52 |
| sex | 0.00 | 0.991 | 65.30 |
| year | 3.75 | 0.026 | 126.93 |
| season | 3.74 | 0.011 | 488.54 |
| PA centered | 11.70 | 0.001 | 42.33 |
| season : PA centered | 1.76 | 0.156 | 268.05 |

## Season × PA on Calmness

**Table 5***Multilevel results for Calmness*

| Predictors | Estimates | std. Error | CI | t | p | df |
| --- | --- | --- | --- | --- | --- | --- |
| (Intercept) | 69.23 | 2.57 | 64.16 – 74.29 | 26.98 | **<0.001** | 175.77 |
| time centered | 0.24 | 0.03 | 0.17 – 0.30 | 6.74 | **<0.001** | 10559.46 |
| time squared centered | 0.02 | 0.01 | 0.00 – 0.04 | 2.46 | **0.014** | 10666.44 |
| mean PA per person per burst | -1.36 | 2.90 | -7.09 – 4.37 | -0.47 | 0.640 | 172.27 |
| sex [female] | 1.14 | 2.54 | -3.92 – 6.20 | 0.45 | 0.654 | 65.48 |
| year [1] | -1.39 | 1.11 | -3.59 – 0.81 | -1.25 | 0.213 | 137.46 |
| year [2] | -1.84 | 1.06 | -3.94 – 0.26 | -1.73 | 0.085 | 126.08 |
| season [Spring] | 1.12 | 1.72 | -2.26 – 4.49 | 0.65 | 0.516 | 417.59 |
| season [Summer] | 0.54 | 1.53 | -2.47 – 3.55 | 0.35 | 0.726 | 471.48 |
| season [Winter] | 3.93 | 1.22 | 1.53 – 6.33 | 3.21 | **0.001** | 965.55 |
| PA centered | -1.93 | 0.63 | -3.18 – -0.68 | -3.06 | **0.003** | 114.96 |
| season [Spring] × PA centered | 0.05 | 0.86 | -1.64 – 1.75 | 0.06 | 0.950 | 356.49 |
| season [Summer] × PA centered | -0.23 | 0.78 | -1.77 – 1.30 | -0.30 | 0.765 | 600.27 |
| season [Winter] × PA centered | 0.76 | 0.88 | -0.98 – 2.49 | 0.86 | 0.390 | 339.83 |

**Table 6***Type III Analysis of Variance Table with Satterthwaite's method*

| Predictors | F | p | df |
| --- | --- | --- | --- |
| time centered | 45.42 | **<0.001** | 10559.46 |
| time squared centered | 6.05 | **0.014** | 10666.44 |
| mean PA per person per burst | 0.22 | 0.640 | 172.27 |
| sex | 0.20 | 0.654 | 65.48 |
| year | 1.59 | 0.209 | 129.94 |
| season | 3.66 | **0.012** | 445.43 |
| PA centered | 15.44 | **<0.001** | 52.87 |
| season : PA centered | 0.38 | 0.771 | 432.61 |

# Moderation Analyses: Sex

## Sex × PA on Energetic Arousal

**Table 7***Multilevel results for Energetic Arousal*

| Predictors | Estimates | std. Error | CI | t | p | df |
| --- | --- | --- | --- | --- | --- | --- |
| (Intercept) | 62.02 | 2.61 | 56.88 – 67.17 | 23.77 | **<0.001** | 177.48 |
| time centered | -0.55 | 0.04 | -0.63 – -0.47 | -13.54 | **<0.001** | 10570.86 |
| time squared centered | -0.25 | 0.01 | -0.26 – -0.23 | -26.30 | **<0.001** | 10688.38 |
| mean PA per person per burst | 1.83 | 2.93 | -3.96 – 7.61 | 0.62 | 0.534 | 170.52 |
| season [Spring] | 5.28 | 1.81 | 1.72 – 8.84 | 2.92 | **0.004** | 352.19 |
| season [Summer] | 3.05 | 1.62 | -0.14 – 6.25 | 1.88 | 0.061 | 381.53 |
| season [Winter] | 1.20 | 1.33 | -1.42 – 3.82 | 0.90 | 0.367 | 709.82 |
| year [1] | -1.92 | 1.13 | -4.15 – 0.32 | -1.70 | 0.092 | 134.77 |
| year [2] | -1.61 | 1.07 | -3.73 – 0.52 | -1.50 | 0.137 | 123.48 |
| sex [female] | -1.32 | 2.57 | -6.45 – 3.82 | -0.51 | 0.611 | 66.88 |
| PA centered | 3.68 | 0.66 | 2.36 – 5.00 | 5.58 | **<0.001** | 58.81 |
| sex [female] × PA centered | 1.90 | 1.01 | -0.11 – 3.92 | 1.89 | 0.063 | 59.30 |

**Table 8***Type III Analysis of Variance Table with Satterthwaite's method*

| Predictors | F | p | df |
| --- | --- | --- | --- |
| time centered | 183.42 | **<0.001** | 10570.86 |
| time squared centered | 691.93 | **<0.001** | 10688.38 |
| mean PA per person per burst | 0.39 | 0.534 | 170.52 |
| season | 2.92 | **0.034** | 359.79 |
| year | 1.69 | 0.188 | 127.33 |
| sex | 0.26 | 0.611 | 66.88 |
| PA centered | 83.82 | **<0.001** | 60.69 |
| sex : PA centered | 3.59 | 0.063 | 59.30 |

## Sex × PA on Valence

**Table 9***Multilevel results for Valence*

| Predictors | Estimates | std. Error | CI | t | p | df |
| --- | --- | --- | --- | --- | --- | --- |
| (Intercept) | 72.85 | 2.44 | 68.04 – 77.67 | 29.83 | **<0.001** | 177.85 |
| time centered | 0.25 | 0.03 | 0.19 – 0.32 | 7.70 | **<0.001** | 10560.72 |
| time squared centered | -0.02 | 0.01 | -0.03 – -0.00 | -2.42 | **0.015** | 10661.41 |
| mean PA per person per burst | 0.68 | 2.85 | -4.93 – 6.30 | 0.24 | 0.810 | 177.16 |
| season [Spring] | 4.71 | 1.65 | 1.47 – 7.95 | 2.86 | **0.004** | 437.59 |
| season [Summer] | 1.11 | 1.47 | -1.77 – 4.00 | 0.76 | 0.449 | 510.71 |
| season [Winter] | 2.91 | 1.17 | 0.62 – 5.20 | 2.50 | **0.013** | 1075.55 |
| year [1] | -0.78 | 1.12 | -2.99 – 1.42 | -0.70 | 0.483 | 134.17 |
| year [2] | -2.81 | 1.07 | -4.93 – -0.70 | -2.63 | **0.010** | 123.05 |
| sex [female] | 0.03 | 2.31 | -4.59 – 4.65 | 0.01 | 0.991 | 64.39 |
| PA centered | 1.56 | 0.53 | 0.48 – 2.64 | 2.92 | **0.006** | 43.07 |
| sex [female] × PA centered | -0.32 | 0.82 | -1.96 – 1.32 | -0.39 | 0.696 | 43.41 |

**Table 10***Type III Analysis of Variance Table with Satterthwaite's method*

| Predictors | F | p | df |
| --- | --- | --- | --- |
| time centered | 59.34 | **<0.001** | 10560.72 |
| time squared centered | 5.86 | **0.015** | 10661.41 |
| mean PA per person per burst | 0.06 | 0.810 | 177.16 |
| season | 3.78 | **0.011** | 486.17 |
| year | 3.77 | **0.026** | 126.82 |
| sex | 0.00 | 0.991 | 64.39 |
| PA centered | 11.68 | **0.001** | 44.43 |
| sex : PA centered | 0.15 | 0.696 | 43.41 |

## Sex × PA on Calmness

**Table 11***Multilevel results for Calmness*

| Predictors | Estimates | std. Error | CI | t | p | df |
| --- | --- | --- | --- | --- | --- | --- |
| (Intercept) | 69.22 | 2.57 | 64.15 – 74.28 | 26.97 | **<0.001** | 175.72 |
| time centered | 0.23 | 0.03 | 0.17 – 0.30 | 6.71 | **<0.001** | 10560.75 |
| time squared centered | 0.02 | 0.01 | 0.00 – 0.04 | 2.46 | **0.014** | 10667.32 |
| mean PA per person per burst | -1.36 | 2.90 | -7.09 – 4.37 | -0.47 | 0.641 | 172.27 |
| season [Spring] | 1.13 | 1.72 | -2.25 – 4.50 | 0.66 | 0.512 | 417.67 |
| season [Summer] | 0.55 | 1.53 | -2.46 – 3.56 | 0.36 | 0.722 | 471.40 |
| season [Winter] | 3.92 | 1.22 | 1.52 – 6.32 | 3.21 | **0.001** | 965.88 |
| year [1] | -1.39 | 1.11 | -3.59 – 0.81 | -1.25 | 0.213 | 137.45 |
| year [2] | -1.84 | 1.06 | -3.94 – 0.26 | -1.73 | 0.086 | 126.07 |
| sex [female] | 1.16 | 2.54 | -3.91 – 6.22 | 0.46 | 0.650 | 65.47 |
| PA centered | -1.82 | 0.60 | -3.03 – -0.62 | -3.05 | **0.004** | 51.17 |
| sex [female] × PA centered | 0.07 | 0.91 | -1.76 – 1.90 | 0.07 | 0.941 | 51.41 |

**Table 12***Type III Analysis of Variance Table with Satterthwaite's method*

| Predictors | F | p | df |
| --- | --- | --- | --- |
| time centered | 45.06 | **<0.001** | 10560.75 |
| time squared centered | 6.05 | **0.014** | 10667.32 |
| mean PA per person per burst | 0.22 | 0.641 | 172.27 |
| season | 3.65 | **0.013** | 445.47 |
| year | 1.58 | 0.209 | 129.94 |
| sex | 0.21 | 0.650 | 65.47 |
| PA centered | 15.24 | **<0.001** | 52.52 |
| sex : PA centered | 0.01 | 0.941 | 51.41 |

# Moderation Analyses: Age group

## Age group × PA on Energetic Arousal

**Table 13***Multilevel results for Energetic Arousal*

| Predictors | Estimates | std. Error | CI | t | p | df |
| --- | --- | --- | --- | --- | --- | --- |
| (Intercept) | 60.86 | 2.87 | 55.20 – 66.53 | 21.23 | **<0.001** | 145.20 |
| time centered | -0.55 | 0.04 | -0.63 – -0.47 | -13.46 | **<0.001** | 10567.08 |
| time squared centered | -0.25 | 0.01 | -0.26 – -0.23 | -26.23 | **<0.001** | 10678.43 |
| mean PA per person per burst | 1.66 | 2.93 | -4.12 – 7.44 | 0.57 | 0.571 | 170.60 |
| season [Spring] | 5.30 | 1.81 | 1.75 – 8.86 | 2.93 | **0.004** | 351.73 |
| season [Summer] | 3.13 | 1.62 | -0.06 – 6.33 | 1.93 | 0.054 | 382.02 |
| season [Winter] | 1.15 | 1.33 | -1.47 – 3.77 | 0.86 | 0.389 | 708.44 |
| year [1] | -1.89 | 1.13 | -4.13 – 0.34 | -1.68 | 0.096 | 134.93 |
| year [2] | -1.60 | 1.07 | -3.72 – 0.53 | -1.49 | 0.140 | 123.65 |
| sex [female] | -1.25 | 2.60 | -6.44 – 3.94 | -0.48 | 0.633 | 66.14 |
| age group [adult] | 2.04 | 2.61 | -3.17 – 7.25 | 0.78 | 0.437 | 61.71 |
| PA centered | 2.16 | 0.80 | 0.57 – 3.74 | 2.71 | **0.008** | 78.09 |
| age group [adult] × PA centered | 3.47 | 0.98 | 1.52 – 5.42 | 3.55 | **0.001** | 66.23 |

**Table 14***Type III Analysis of Variance Table with Satterthwaite's method*

| Predictors | F | p | df |
| --- | --- | --- | --- |
| time centered | 181.28 | <0.001 | 10567.08 |
| time squared centered | 688.18 | <0.001 | 10678.43 |
| mean PA per person per burst | 0.32 | 0.571 | 170.60 |
| season | 2.98 | 0.031 | 359.86 |
| year | 1.66 | 0.194 | 127.50 |
| sex | 0.23 | 0.633 | 66.14 |
| age group | 0.61 | 0.437 | 61.71 |
| PA centered | 62.85 | <0.001 | 67.32 |
| age group : PA centered | 12.63 | **0.001** | 66.23 |

## Age group × PA on Valence

**Table 15***Multilevel results for Valence*

| Predictors | Estimates | std. Error | CI | t | p | df |
| --- | --- | --- | --- | --- | --- | --- |
| (Intercept) | 74.53 | 2.63 | 69.33 – 79.73 | 28.31 | **<0.001** | 147.28 |
| time centered | 0.25 | 0.03 | 0.19 – 0.32 | 7.65 | **<0.001** | 10556.14 |
| time squared centered | -0.02 | 0.01 | -0.03 – -0.00 | -2.41 | **0.016** | 10649.10 |
| mean PA per person per burst | 0.96 | 2.84 | -4.64 – 6.57 | 0.34 | 0.734 | 177.77 |
| season [Spring] | 4.73 | 1.64 | 1.50 – 7.96 | 2.88 | **0.004** | 433.27 |
| season [Summer] | 1.01 | 1.47 | -1.87 – 3.89 | 0.69 | 0.491 | 507.38 |
| season [Winter] | 2.99 | 1.17 | 0.70 – 5.28 | 2.57 | **0.010** | 1067.34 |
| year [1] | -0.83 | 1.12 | -3.03 – 1.38 | -0.74 | 0.460 | 133.69 |
| year [2] | -2.83 | 1.07 | -4.95 – -0.72 | -2.65 | **0.009** | 122.58 |
| sex [female] | 0.81 | 2.28 | -3.73 – 5.36 | 0.36 | 0.722 | 63.63 |
| age group [adult] | -3.69 | 2.30 | -8.28 – 0.91 | -1.61 | 0.114 | 58.80 |
| PA centered | 1.86 | 0.69 | 0.48 – 3.24 | 2.70 | **0.009** | 59.05 |
| age group [adult] × PA centered | -0.62 | 0.85 | -2.33 – 1.09 | -0.72 | 0.472 | 50.60 |

**Table 16***Type III Analysis of Variance Table with Satterthwaite's method*

| Predictors | F | p | df |
| --- | --- | --- | --- |
| time centered | 58.57 | **<0.001** | 10556.14 |
| time squared centered | 5.82 | **0.016** | 10649.10 |
| mean PA per person per burst | 0.12 | 0.734 | 177.77 |
| season | 3.95 | **0.008** | 484.17 |
| year | 3.79 | **0.025** | 126.36 |
| sex | 0.13 | 0.722 | 63.63 |
| age group | 2.58 | 0.114 | 58.80 |
| PA centered | 13.15 | **0.001** | 51.41 |
| age group : PA centered | 0.53 | 0.472 | 50.60 |

## Age group × PA on Calmness

**Table 17***Multilevel results for Calmness*

| Predictors | Estimates | std. Error | CI | t | p | df |
| --- | --- | --- | --- | --- | --- | --- |
| (Intercept) | 67.85 | 2.82 | 62.28 – 73.42 | 24.07 | **<0.001** | 142.32 |
| time centered | 0.24 | 0.03 | 0.17 – 0.30 | 6.72 | **<0.001** | 10556.48 |
| time squared centered | 0.02 | 0.01 | 0.00 – 0.04 | 2.41 | **0.016** | 10653.78 |
| mean PA per person per burst | -1.46 | 2.90 | -7.19 – 4.27 | -0.50 | 0.616 | 172.11 |
| season [Spring] | 1.09 | 1.72 | -2.29 – 4.46 | 0.63 | 0.526 | 416.79 |
| season [Summer] | 0.56 | 1.53 | -2.45 – 3.57 | 0.36 | 0.716 | 470.76 |
| season [Winter] | 3.87 | 1.22 | 1.47 – 6.28 | 3.17 | **0.002** | 964.66 |
| year [1] | -1.37 | 1.11 | -3.57 – 0.83 | -1.23 | 0.220 | 137.54 |
| year [2] | -1.83 | 1.06 | -3.93 – 0.27 | -1.73 | 0.087 | 126.17 |
| sex [female] | 0.44 | 2.60 | -4.75 – 5.63 | 0.17 | 0.865 | 64.56 |
| age group [adult] | 2.99 | 2.56 | -2.13 – 8.11 | 1.17 | 0.248 | 60.37 |
| PA centered | -1.37 | 0.77 | -2.90 – 0.16 | -1.79 | 0.078 | 68.33 |
| age group [adult] × PA centered | -0.66 | 0.95 | -2.55 – 1.24 | -0.69 | 0.492 | 58.73 |

**Table 18***Type III Analysis of Variance Table with Satterthwaite's method*

| Predictors | F | p | df |
| --- | --- | --- | --- |
| time centered | 45.20 | **<0.001** | 10556.48 |
| time squared centered | 5.81 | **0.016** | 10653.78 |
| mean PA per person per burst | 0.25 | 0.616 | 172.11 |
| season | 3.56 | **0.014** | 445.12 |
| year | 1.57 | 0.213 | 130.03 |
| sex | 0.03 | 0.865 | 64.56 |
| age group | 1.36 | 0.248 | 60.37 |
| PA centered | 12.74 | **0.001** | 59.56 |
| age group : PA centered | 0.48 | 0.492 | 58.73 |
